# Supplementary material for: New light shed on the early evolution of limb-bone growth plate and bone marrow
Source: eLife. 2021 Mar 2;10:e51581. doi: 10.7554/eLife.51581 (PMC7924947; doi:10.7554/eLife.51581)
Supplement: Transparent reporting form [file elife-51581-transrepform.docx]

***eLife’s* transparent reporting form**

We encourage authors to provide detailed information *within their submission* to facilitate the interpretation and replication of experiments. Authors can upload supporting documentation to indicate the use of appropriate reporting guidelines for health-related research (see [EQUATOR Network](http://www.equator-network.org/%20)), life science research (see the [BioSharing Information Resource](https://biosharing.org/)), or the [ARRIVE guidelines](http://www.plosbiology.org/article/info:doi/10.1371/journal.pbio.1000412) for reporting work involving animal research. Where applicable, authors should refer to any relevant reporting standards documents in this form.

If you have any questions, please consult our Journal Policies and/or contact us: [editorial@elifesciences.org](mailto:editorial@elifesciences.org).

**Sample-size estimation**

- You should state whether an appropriate sample size was computed when the study was being designed
- You should state the statistical method of sample size computation and any required assumptions
- If no explicit power analysis was used, you should describe how you decided what sample (replicate) size (number) to use

Please outline where this information can be found within the submission (e.g., sections or figure legends), or explain why this information doesn’t apply to your submission:

All fossil specimens come from museum/university collections. We describe in the material section where they come from (locality and geological time period) as well as where they are hosted (collections). Their collection reference number is provided in the material section as well as in the result section. When possible, we sectioned/scanned juvenile and adult individuals of the same species. When not, we only scanned (almost) mature individuals. One individual per developmental stage per species was investigated because synchrotron scanning is very costly and 3D segmentation greatly time-consuming. This is very common in vertebrate paleontology. We invested time in considering several taxa within the group of temnospondyls and within the group of seymouriamorphs to strengthen our hypothesis.

**Replicates**

- You should report how often each experiment was performed
- You should include a definition of biological versus technical replication
- The data obtained should be provided and sufficient information should be provided to indicate the number of independent biological and/or technical replicates
- If you encountered any outliers, you should describe how these were handled
- Criteria for exclusion/inclusion of data should be clearly stated
- High-throughput sequence data should be uploaded before submission, with a private link for reviewers provided (these are available from both GEO and ArrayExpress)

Please outline where this information can be found within the submission (e.g., sections or figure legends), or explain why this information doesn’t apply to your submission:

Our study focuses on fossil samples, for that reason, no replicate is possible. Whenever possible, we sectioned/scanned several individuals of the same species (see explanations above). Thin sections are deposited in the same institutions as the fossil bones and can be observed by anyone who would like to check the pictures we took to produce our figures. Scan data of the regions of interest will be deposited on the palaeo-database (http://paleo.esrf.eu/) of the European Synchrotron Radiation Facility (ESRF) so that anyone can check our segmentation.

**Statistical reporting**

- Statistical analysis methods should be described and justified
- Raw data should be presented in figures whenever informative to do so (typically when N per group is less than 10)
- For each experiment, you should identify the statistical tests used, exact values of N, definitions of center, methods of multiple test correction, and dispersion and precision measures (e.g., mean, median, SD, SEM, confidence intervals; and, for the major substantive results, a measure of effect size (e.g., Pearson's r, Cohen's d)
- Report exact p-values wherever possible alongside the summary statistics and 95% confidence intervals. These should be reported for all key questions and not only when the p-value is less than 0.05.

Please outline where this information can be found within the submission (e.g., sections or figure legends), or explain why this information doesn’t apply to your submission:

No statistics could be made on our data. Our study is based on microstructural observations. We provided images of the raw data as well as images of our 3D models so that the reader can check our segmentation interpretation for Fig. 6 to 10 and 12. Figs. 2-5 are pictures of the original fossil thin sections.

(For large datasets, or papers with a very large number of statistical tests, you may upload a single table file with tests, Ns, etc., with reference to sections in the manuscript.)

**Group allocation**

- Indicate how samples were allocated into experimental groups (in the case of clinical studies, please specify allocation to treatment method); if randomization was used, please also state if restricted randomization was applied
- Indicate if masking was used during group allocation, data collection and/or data analysis

Please outline where this information can be found within the submission (e.g., sections or figure legends), or explain why this information doesn’t apply to your submission:

We selected two species of temnospondyls (i.e. stem batrachians) and two species of seymouriamorphs (i.e. stem amniotes). Information from stem tetrapods and extant groups of tetrapods were provided from the literature. All this was stated in the introduction, as well as in the material section. All references to the cited articles were included in the reference list.

**Additional data files (“source data”)**

- We encourage you to upload relevant additional data files, such as numerical data that are represented as a graph in a figure, or as a summary table
- Where provided, these should be in the most useful format, and they can be uploaded as “Source data” files linked to a main figure or table
- Include model definition files including the full list of parameters used
- Include code used for data analysis (e.g., R, MatLab)
- Avoid stating that data files are “available upon request”

Please indicate the figures or tables for which source data files have been provided:

Raw data are already provided for each sample in the figures of the main text. Fossils and thin sections are all available in museum/university collections. Regions of interest of the scan data will be uploaded on the ESRF palaeo-database of the ESRF which is accessible to anyone.
